# Comparison of the efficacy of single vs. double autologous platelet-rich plasma intrauterine infusion on endometrial receptivity in thin endometrium patients: a prospective randomized controlled trial

**DOI:** 10.3389/fendo.2025.1609556

**Published:** 2025-08-28

**Authors:** Keng Feng, Lingling Zhu, Yudi Luo, Luhai Ruan, Derong Li, Xiang Li, Ling Li, Ling Wu

**Affiliations:** Center of Reproductive Medicine, Yulin Maternal and Child Health Hospital, Yulin, China

**Keywords:** thin endometrium, platelet-rich plasma, double intrauterine infusion, endometrial receptivity, treatment

## Abstract

**Objective:**

To compare single versus double autologous platelet-rich plasma (PRP) intrauterine infusion effects on endometrial receptivity and pregnancy outcomes in patients with thin endometrium.

**Methods:**

This randomized controlled trial included 100 patients with thin endometrium and infertility, assigned to single (n=50) or double (n=50) infusion groups. The single group received 1.0 ml of PRP on day 11 of the hormone replacement therapy-frozen embryo transfer (HRT-FET) cycle, while the double group received 1.0 ml on both days 11 and 13. Primary outcomes included endometrial thickness, receptivity changes, and clinical pregnancy rates; secondary outcomes were cycle cancellation rate, miscarriage rate, and endometrial hemodynamics. Statistical analysis was conducted using SPSS 26.0.

**Results:**

The double PRP infusion group exhibited measurable improvements in endometrial and early pregnancy outcomes. 1) Endometrial thickness increased (8.42 ± 0.53 mm vs 7.96 ± 0.45 mm, P<0.01); 2) Hemodynamic parameters improved for resistance index (RI) (1.72 ± 0.08 vs 1.79 ± 0.08, P<0.01) and pulsatility index (PI) (3.83 ± 0.64 vs 4.38 ± 0.68, P<0.01); 3) Clinical outcomes: lower cycle cancellation rate (10.0% vs 26.0%, P=0.037) and higher clinical pregnancy rate (48.9% vs 27.0%, P=0.043).However, early miscarriage rates were similar between groups (p > 0.99).

**Conclusion:**

Compared to a single intrauterine infusion, double intrauterine PRP infusion may enhance the receptivity of a thin endometrium and improve clinical pregnancy outcomes. However, since the study population did not include patients with thin endometrium who also have a history of recurrent implantation failure or recurrent miscarriage, caution is advised when applying these findings to this specific group. Furthermore, these conclusions require validation through larger, randomized, multicenter trials.

## Introduction

Thin endometrium, defined as insufficient endometrial thickness during the ovulatory phase or implantation window, is a pathological condition often associated with poor reproductive outcomes. Current research generally defines thin endometrium as an endometrial thickness of less than 7 mm during embryo transfer (1, 2). Clinical pregnancy rates are notably low, approximately 29.43%, when the endometrial thickness is ≤6 mm at the time of transfer (3). Therefore, improving the thickness and functionality of the endometrium is considered a crucial strategy for enhancing reproductive success. However, the underlying pathological mechanisms of thin endometrium remain unclear. Some studies suggest that basal layer damage, leading to extracellular matrix and collagen fiber deposition, which subsequently results in endometrial fibrosis, could be a potential cause (4). Current treatment options for thin endometrium include increasing estrogen dosage, using sildenafil, intrauterine infusion of granulocyte colony-stimulating factor (G-CSF), and application of peripheral blood mononuclear cells (1, 5, 6). However, these treatments have not shown satisfactory results in clinical practice, highlighting the importance of exploring new therapeutic approaches.

Platelet-Rich Plasma (PRP), obtained by centrifuging autologous whole blood, is a high-concentration platelet preparation, with a platelet concentration 4 to 6 times higher than normal blood (7). PRP is rich in various growth factors, including Platelet-Derived Growth Factor (PDGF), Vascular Endothelial Growth Factor (VEGF), and Insulin-like Growth Factor 1 (IGF-1) (8). These growth factors promote endometrial cell proliferation, angiogenesis, and antifibrosis, which makes PRP a widely studied option for tissue repair and regeneration. In recent years, the use of PRP in patients with thin endometrium has garnered increasing attention. Multiple clinical studies have shown that PRP intrauterine infusion can significantly increase endometrial thickness in patients with thin endometrium (9, 10). However, most existing studies focus on single infusion protocols, with limited research on the efficacy of multiple infusions. Unresolved issues include: 1) whether multiple infusions can further improve patient outcomes; 2) the optimal interval between repeated infusions; 3) the dose-response relationship between platelet concentration and clinical outcomes. These issues limit the widespread clinical application of PRP.

Thus, this study aims to compare the effects of single versus double PRP intrauterine infusion in a randomized controlled trial (RCT) design to provide stronger evidence for individualized treatment of patients with thin endometrium. The results of this study will provide important guidance for clinical practice and contribute to the better management and treatment of infertility associated with thin endometrium.

## Study design

### Sample size estimation

Sample size calculation in this study was performed using G*Power 3.1 software, based on previously reported differences (11–13) in endometrial thickness following single versus double intrauterine infusions of PRP (single infusion group: 7.21 ± 0.18 mm, double infusion group: 5.99 ± 0.70 mm) and clinical pregnancy rates (single infusion group: 44.12%, double infusion group: 22.7%). The significance level (one-sided α) was set at 0.05, with a statistical power of 90% and an allocation ratio of 1:1 between the two groups. The calculated sample size was 42 participants per group. Considering potential covariate adjustments and possible dropout, the final sample size was set at 50 participants per group (total n = 100).

### Randomization and blinding

**Figure 1:**
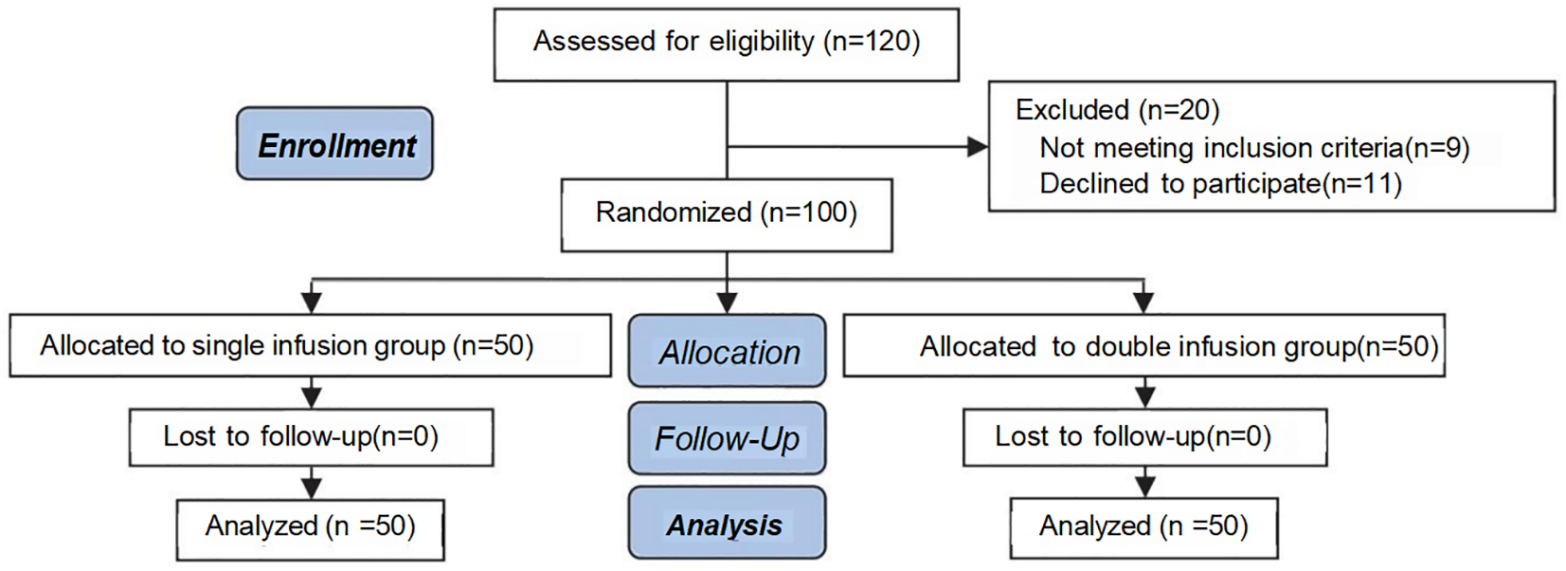
Consort flow diagram.

This study is a single-blinded, randomized controlled trial conducted at the Reproductive Center of Yulin Maternal and Child Health Hospital from July 2023 to February 2025. A total of 120 patients with thin endometrium were screened, and 100 subjects who met strict inclusion and exclusion criteria were ultimately enrolled (reasons for exclusion: 9 did not meet the inclusion criteria and 11 declined participation). No subjects were lost to follow-up during the study period (see **Figure 1**). A computer-generated randomization sequence was produced using the Chinese Clinical Trial Registry system, and central randomization allocated participants in a 1:1 ratio to either the single infusion group (n=50) or the double infusion group (n=50).

A single-blind design was implemented: participants were unaware of their group assignment. All ultrasound assessments were performed by independent radiologists who were blinded to group allocation, and strict separation was maintained between the investigators and the imaging physicians to minimize assessment bias.

The intervention protocol was as follows: All enrolled participants underwent standard hormone replacement therapy (HRT) for frozen embryo transfer (FET) cycles. In the single infusion group, 1.0 ml of autologous platelet-rich plasma (PRP) was infused intrauterinely on day 11 of the cycle, followed by an identical volume of saline on day 13. In the double infusion group, 1.0 ml of autologous PRP was infused intrauterinely on both day 11 and day 13 of the cycle.

#### Inclusion criteria

①Female patients aged between 25 and 40 years ②Diagnosis of thin endometrium, defined as an endometrial thickness of less than 7 mm on the day of trigger in the IVF cycle or on the endometrial transformation day in the FET cycle;③Availability of at least one high-quality blastocyst for transfer. The embryo grading method used in our center follows the Gardner blastocyst grading system (14). High-quality blastocysts are defined as those with a developmental stage of ≥3 and an inner cell mass and/or trophectoderm grade of A and/or B.

#### Exclusion criteria

①Patients with a history of recurrent implantation failure or recurrent miscarriage;②Presence of significant uterine anomalies (e.g., unicornuate uterus, septate uterus);③Severe endometriosis or adenomyosis;④Severe intrauterine adhesions;⑤One partner having chromosomal abnormalities;⑥Coexisting pregnancy-related autoimmune diseases, as well as infectious diseases such as hepatitis, tuberculosis, and syphilis;⑦Patients with thrombocytopenia or thrombocytosis. Patients meeting any of the above exclusion criteria will not be included in this study.

### Research methods

#### Endometrial preparation protocol

The hormone replacement regimen consists of administering estradiol valerate at a dosage of 3 mg, twice daily, starting on the second day of the menstrual cycle. A transvaginal ultrasound will be performed on day 11 of the menstrual cycle to measure endometrial thickness at the thickest point in the sagittal plane of the uterus. Measurements will be taken by the same physician twice, and the average of the two measurements will be recorded. If the endometrial thickness is ≥7 mm, the patient will be excluded from the study, and subsequent medication will be adjusted accordingly.

#### Preparation of PRP and intrauterine infusion

①After informing the patient of the associated risks and obtaining informed consent, a comprehensive pre-collection assessment will be performed, including vital signs, complete blood count (with a requirement for platelet count ≥150 × 10^9/L), coagulation profile, and serological tests for hepatitis B surface antigen, hepatitis C antibodies, syphilis antibodies, and HIV antibodies;

②In a 10 ml syringe, 1 ml of sodium citrate anticoagulant will be added, followed by the collection of 8 ml of peripheral venous blood. PRP will be prepared using the two-step centrifugation method as described by Landesberg (15). Blood specimens will be evenly distributed into two 5 ml centrifuge tubes and centrifuged at 200 × g for 15 minutes. After centrifugation, three layers will be observed: the plasma layer, the platelet-leukocyte layer, and the red blood cell layer. The plasma and platelet-leukocyte layers will be collected into another 5 ml centrifuge tube and centrifuged again at 300 × g for 10 minutes. After the second centrifugation, the bottom 1.1 ml of liquid will be collected as PRP, with 0.1 ml reserved for platelet concentration testing, which should be 4 to 5 times the patient’s plasma concentration;

③Activation of PRP: To each milliliter of PRP, 0.2 ml of 10% CaCl2 buffer will be added and mixed thoroughly, followed by the addition of 10 U bovine thrombin per milliliter of PRP. The mixture will then be placed in a 37°C incubator for 1 minute to activate and achieve a gel state. Activated PRP must be infused within 1 hour;

④Infusion: The patient will void their bladder and assume the lithotomy position. Sterilization and saline irrigation will be performed prior to the procedure. Under ultrasound guidance, an embryo transfer catheter will be inserted, and 1 ml of the PRP solution will be slowly infused.

#### Observational parameters

Doppler ultrasound will be utilized for transvaginal monitoring of endometrial status. Measurements will be taken on the endometrial transformation day using a three-dimensional probe. Parameters will include: endometrial thickness, endometrial volume, endometrial blood flow grading (I, II, III types), bilateral uterine artery pulsatility index (PI), bilateral resistance index (RI), and the ratio of peak systolic to end-diastolic flow velocities (S/D). Pregnancy-related parameters will include clinical pregnancy (defined as the visualization of fetal heartbeat or gestational sac via ultrasound 28–35 days post-transfer), clinical pregnancy rate (number of clinical pregnancies/number of patients undergoing embryo transfer), and early miscarriage rate (number of miscarriages before 12 weeks/number of clinical pregnancies).

#### Luteal support

Following embryo transfer, patients will receive either 400 mg/day of oral progesterone capsules, 40 mg/day of intramuscular progesterone injection, or 200 mg/day of vaginal progesterone soft capsules. Luteal support will continue until 12 weeks of gestation.

#### Study ethics

This study is a single-center, prospective randomized controlled trial (RCT). It has been registered with the Chinese Clinical Trial Registry (Registration No.: ChiCTR2300071473) and approved by the Ethics Committee of Yulin Maternal and Child Health Hospital (Approval No.: YLSFYLL2023-01-06-09).

### Statistical analysis

Data from this study will be analyzed using SPSS version 26.0 statistical software. Normally distributed continuous variables will be described as mean ± standard deviation (± s), and inter-group comparisons will be conducted using t-tests. For non-normally distributed continuous variables, data will be described as median (25th percentile, 75th percentile) [M (P25, P75)], with inter-group comparisons performed using non-parametric tests. Categorical data will be presented as counts (percentages) [n (%)], with inter-group comparisons conducted using the chi-square test.

## Results

### Comparison of baseline characteristics

**Table 1** presents a comparison of the baseline characteristics between the single infusion group and the double infusion group. The analysis reveals no significant differences between the two groups in the following key parameters: female age, male age, duration of infertility, body mass index (BMI), anti-Müllerian hormone (AMH) levels, serum platelet concentration, platelet concentration in platelet-rich plasma (PRP), and estradiol (E2) levels (p > 0.05). These results indicate that the two groups were highly comparable in terms of baseline characteristics, providing a solid foundation for the subsequent comparison of clinical outcomes.

**Table 1:** Comparison of baseline characteristics.

Characteristic	Single infusion group, N = 50	Double infusion group, N = 50	t	P-value
Female Age (year)	30.7 ± 3.6	29.6 ± 3.6	1.484	0.141
Male Age (year)	32.54 ± 3.02	33.58 ± 3.04	-1.717	0.089
Duration of Infertility (year)	3.91 ± 2.42	4.47 ± 2.40	-1.163	0.248
BMI	22.96 ± 3.12	22.37 ± 2.86	0.995	0.322
AMH	4.23 ± 2.83	3.52 ± 3.00	1.219	0.226
Platelet Concentration in Serum	313 ± 26	300 ± 25	2.422	0.017
Platelet Concentration in PRP	1,406 ± 118	1,398 ± 115	0.356	0.723
Estradiol Level (E2)	237 ± 34	250 ± 34	-1.811	0.073

### Comparison of endometrial receptivity

**Table 2** presents a comparison of endometrial receptivity between the two patient groups. The analysis indicates that patients in the double infusion group exhibited significantly greater endometrial thickness (8.42 ± 0.53 mm vs. 7.96 ± 0.45 mm, P < 0.01) and a higher increase in thickness (2.19 ± 0.34 mm vs. 1.74 ± 0.34 mm, P < 0.01) compared to those in the single infusion group. Additionally, the endometrial volume in the double infusion group was also significantly larger (3.26 ± 0.59 mm vs. 2.89 ± 0.76 mm, P < 0.01), with notable improvements in blood flow parameters. Further hemodynamic analysis revealed that the pulsatility index (PI) of the bilateral uterine arteries was significantly lower in the double infusion group (3.83 ± 0.64 vs. 4.38 ± 0.68, P < 0.01), as was the resistance index (RI) (1.72 ± 0.08 vs. 1.79 ± 0.08, P < 0.01), and the systolic-to-diastolic blood flow velocity ratio (S/D, 10.50 ± 1.21 vs. 11.70 ± 1.22, P < 0.01). These findings collectively suggest a marked improvement in endometrial receptivity among patients in the double infusion group, indicating that this intervention may positively influence the chances of conception.

**Table 2:** Comparison of endometrial receptivity.

Characteristic	Single infusion group, N = 50	Double infusion group, N = 50	t/χ2	P-value
Endometrial Thickness Before Infusion (mm)	6.22 ± 0.51	6.03 ± 0.48	1.928	0.057
Endometrial Thickness After Infusion (mm)	7.96 ± 0.45	8.42 ± 0.53	-4.676	<0.001
Endometrial Growth Value (mm)	1.74 ± 0.34	2.19 ± 0.34	-6.678	<0.001
Endometrial Volume	2.89 ± 0.76	3.26 ± 0.59	-2.734	0.008
Bilateral Uterine Endometrial Vascular Resistance Index (RI)	1.79 ± 0.08	1.72 ± 0.08	4.286	<0.001
Bilateral Pulsatility Index (PI)	4.38 ± 0.68	3.83 ± 0.64	4.165	<0.001
Bilateral Systolic/Diastolic Blood Flow Velocity Ratio (S/D)	11.70 ± 1.22	10.50 ± 1.21	4.948	<0.001
Endometrial Blood Flow Grade			11.20	0.004
I	19 (38.0%)	5 (10.0%)		
II	21 (42.0%)	27 (54.0%)		
III	10 (20.0%)	18 (36.0%)		

### Clinical outcome comparison

**Table 3** summarizes the comparative results of clinical outcomes between the two groups. This study compared the differences between the single infusion group and the double infusion group in terms of cycle cancellation rate, clinical pregnancy rate, and early miscarriage rate. The main findings are as follows: Cycle cancellation rate: The double infusion group demonstrated a significantly lower cycle cancellation rate (10.0%) compared to the single infusion group (26.0%) (OR = 3.16, 95% CI: 1.03–9.68, p = 0.037).Clinical pregnancy rate: The clinical pregnancy rate was significantly higher in the double infusion group (48.9%) than in the single infusion group (27.0%) (OR = 2.58, 95% CI: 1.02–6.56, p = 0.043).Early miscarriage rate: There was no significant difference between the two groups in terms of early miscarriage rate. Although the differences in cycle cancellation rate and clinical pregnancy rate between the groups reached statistical significance (p < 0.05), it is important to note that the lower limits of the 95% confidence intervals for the odds ratios were close to 1.0 (1.03 and 1.02, respectively), indicating a degree of uncertainty in these results. Potential contributing factors may include the limited sample size, unstable effect estimates, and possible residual confounding. In summary, our findings suggest that double infusion may be superior to single infusion in reducing the cycle cancellation rate and improving the clinical pregnancy rate. However, given the relatively wide confidence intervals, larger sample sizes and higher-quality prospective studies are warranted to confirm these results. While our findings may serve as a reference for clinical decision-making, their interpretation and application should be undertaken with caution, in light of the inherent uncertainty and limitations.

**Table 3:** Clinical outcome comparison.

Characteristic	Single infusion group, N = 50	Double infusion group, N = 50	OR(95%CI)	p-value
Cycle cancellation			3.2 (1.03-9.68)	0.037
NO	37 (74.0%)	45 (90.0%)		
YES	13 (26.0%)	5 (10.0%)		
Clinical pregnancy			2.6 (1.02-6.56)	0.043
YES	10 (27.0%)	22 (48.9%)		
NO	27 (73.0%)	23 (51.1%)		
Early miscarriage			0.6 (0.05-6.86)	>0.999
NO	36 (97.3%)	43 (95.6%)		
YES	1 (2.7%)	2 (4.4%)		

### Logistic regression

**Figure 2:**
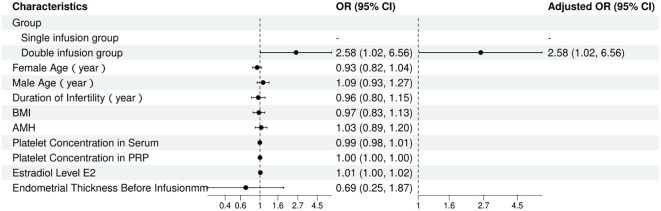
Forest plot of multivariate logistic regression analysis.

Given that potential confounding factors such as age, AMH, and BMI may affect endometrial receptivity and consequently influence clinical pregnancy outcomes, this study employed logistic regression analysis to adjust for these relevant covariates and assess whether double PRP infusion is an independent predictor of clinical pregnancy. **Table 4**; **Figure 2** present the results of the multivariate regression analysis, demonstrating that there were significant differences in clinical pregnancy rates among the different infusion protocols. Compared to the single infusion group, the double infusion group had significantly higher odds of clinical pregnancy (adjusted OR, 2.58; 95% CI, 1.02–6.56; p = 0.046).

**Table 4:** Univariate and multivariate analysis of influencing factors (logistic regression).

Characteristic	Univariable					Multivariable				
	N	Event N	OR	95% CI	p-value	N	Event N	OR	95% CI	p-value
Group										
Single infusion group	37	10	—	—		37	10	—	—	
Double infusion group	45	22	2.58	1.02, 6.56	0.046	45	22	2.58	1.02, 6.56	0.046
Female Age (year)	82	32	0.93	0.82, 1.04	0.210					
Male Age (year)	82	32	1.09	0.93, 1.27	0.307					
Duration of Infertility (year)	82	32	0.96	0.80, 1.15	0.629					
BMI	82	32	0.97	0.83, 1.13	0.705					
AMH	82	32	1.03	0.89, 1.20	0.695					
Platelet Concentration in Serum	82	32	0.99	0.98, 1.01	0.519					
Platelet Concentration in PRP	82	32	1.00	1.00, 1.00	0.790					
Estradiol Level E2	82	32	1.01	1.00, 1.02	0.182					
Endometrial Thickness Before Infusion mm	82	32	0.69	0.25, 1.87	0.463					

CI, Confidence Interval; OR, Odds Ratio.

### Subgroup analysis of early miscarriage rate

As shown in **Table 5**, a comparison was performed between patients who experienced early miscarriage (n=3) and those who did not (n=79) with respect to baseline characteristics. The results demonstrated no statistically significant differences between the two groups in terms of female age (29.3 ± 2.9 years vs. 29.9 ± 3.8 years, p=0.785), male age (35.00 ± 3.00 years vs. 33.18 ± 2.92 years, p=0.403), duration of infertility (5.00 ± 2.29 years vs. 4.12 ± 2.54 years, p=0.578), body mass index (BMI) (22.20 ± 0.31 vs. 22.57 ± 2.96, p=0.332), anti-Müllerian hormone (AMH) level (6.04 ± 8.56 ng/mL vs. 3.64 ± 2.57 ng/mL, p=0.676), serum platelet concentration (310 ± 26 × 10^9/L vs. 306 ± 28 × 10^9/L, p=0.853), PRP platelet concentration (1,426 ± 141 × 10^9/L vs. 1,405 ± 120 × 10^9/L, p=0.826), estradiol level (252 ± 31 pg/mL vs. 245 ± 35 pg/mL, p=0.746), and endometrial thickness on the day of embryo transfer (6.27 ± 0.50 mm vs. 6.22 ± 0.45 mm, p=0.886). Furthermore, there was no significant difference in the distribution of single PRP infusion (33.3% vs. 45.6%) and double PRP infusion (66.7% vs. 54.4%) between the two groups (p>0.999).The lack of a significant difference in early miscarriage rates may be attributed to the fact that early miscarriage is predominantly associated with embryonic chromosomal abnormalities. In this study, the two groups were well-matched in terms of baseline characteristics, and each patient received a single transfer of a high-quality blastocyst, which helped control for relevant confounding factors. Therefore, no statistically significant difference in early miscarriage rates was observed between the groups.

**Table 5:** Subgroup analysis of early miscarriage rate.

Characteristic	Early miscarriage			
	NO N = 79	YES N = 3	t/χ2	p-value
Female Age (year), Mean ± SD	29.9 ± 3.8	29.3 ± 2.9	0.31	0.785^1^
Male Age (year), Mean ± SD	33.18 ± 2.92	35.00 ± 3.00	-1.03	0.403^1^
Duration of Infertility (year), Mean ± SD	4.12 ± 2.54	5.00 ± 2.29	-0.65	0.578^1^
BMI, Mean ± SD	22.57 ± 2.96	22.20 ± 0.31	0.99	0.332^1^
AMH, Mean ± SD	3.64 ± 2.57	6.04 ± 8.56	-0.48	0.676^1^
Platelet Concentration in Serum, Mean ± SD	306 ± 28	310 ± 26	-0.21	0.853^1^
Platelet Concentration in PRP, Mean ± SD	1,405 ± 120	1,426 ± 141	-0.25	0.826^1^
Estradiol Level (E2), Mean ± SD	245 ± 35	252 ± 31	-0.37	0.746^1^
Endometrial Thickness Before Infusion (mm), Mean ± SD	6.22 ± 0.45	6.27 ± 0.50	-0.16	0.886^1^
Group, n (%)				>0.999^2^
Single infusion group	36 (45.6%)	1 (33.3%)		
Double infusion group	43 (54.4%)	2 (66.7%)		

^1^Welch Two Sample t-test.

^2^Fisher’s exact test.

## Discussion

In assisted reproductive technology (ART), successful implantation and live birth are influenced by multiple factors, with endometrial receptivity and embryo quality being the most critical determinants (16). Evidence indicates that approximately two-thirds of embryo transfer failures are associated with impaired endometrial receptivity (17). Both endometrial thickness and blood flow parameters, serving as key ultrasonographic markers of endometrial receptivity, significantly influence post-transfer pregnancy outcomes (1). Thin endometrium, defined as an endometrial thickness below the threshold necessary to support pregnancy, affects approximately 2.4% of women with infertility (18). Studies suggest that a thin endometrium may contribute to recurrent implantation failure, cancellation of *in vitro* fertilization-embryo transfer (IVF-ET) cycles, and an elevated risk of obstetric complications such as preeclampsia and placental abruption, thereby substantially reducing both clinical pregnancy rates and live birth rates (19, 20). Platelet-rich plasma (PRP), a concentrated autologous plasma preparation obtained via sequential gradient centrifugation, contains high platelet levels that release a plethora of growth factors and cytokines upon activation, promoting tissue regeneration and repair (21). Given its autologous nature, PRP exhibits a favorable safety profile with minimal adverse effects, making it widely applicable in clinical practice—particularly in orthopedic therapies and wound healing applications (22–25). In 2015, Chang et al (26) first reported the successful use of intrauterine PRP infusion in women with thin endometrium, demonstrating improved endometrial parameters and achieving clinical pregnancy. This breakthrough introduced a novel therapeutic strategy for enhancing reproductive outcomes in patients with thin endometrium. Subsequently, there has been growing interest in exploring the potential role of PRP in optimizing endometrial receptivity within ART protocols.

Despite growing clinical interest, robust evidence regarding the optimal number of platelet-rich plasma (PRP) intrauterine infusions remains limited, with no evidence-based consensus established to date. From a molecular perspective, platelet-derived growth factors exhibit short biological half-lives: VEGF demonstrates a half-life of <30 minutes, while PDGF and FGF have half-lives of 2.4 hours and 7.6 hours, respectively (27, 28). This pharmacokinetic profile suggests that a single infusion may fail to sustain therapeutic concentrations over the critical endometrial receptivity window. Notably, Kobayashi et al (29) provided further mechanistic insight into PRP’s growth factor release kinetics, reporting a rapid PDGF surge within 0–15 minutes post-infusion, followed by a sustained low-level release phase—a finding supporting the rationale for sequential administration. This is corroborated by Nazari et al (13) and Eftekhar et al (30), whose series of studies demonstrated that in patients with suboptimal endometrial response at 48 hours post-initial infusion, a second PRP administration significantly improved pregnancy outcomes. These findings align with Chang et al.’s (11) recommendation that when using a 0.5–1 mL PRP dose protocol, a repeat infusion should be considered if endometrial parameters remain inadequate within 48 hours. Collectively, these data underscore the potential benefits of a dual-infusion strategy in optimizing endometrial receptivity. To further evaluate this approach, we conducted an RCT enrolling 100 women with thin endometrium randomly allocated to single versus double PRP intrauterine infusion groups. The dual-infusion cohort demonstrated: a significant improvement in endometrial receptivity markers (increased thickness + enhanced vascular perfusion), 16% reduction in cycle cancellation rates, 21.9% increase in clinical pregnancy rates compared to single-infusion controls. Our indings contribute to the growing body of evidence supporting the efficacy of dual PRP infusions, particularly when endometrial assessment at 48 hours indicates suboptimal response. Based on our results and prior mechanistic studies, we advocate for protocol adjustments incorporating a second PRP administration in such cases to maximize therapeutic benefit. Further large-scale trials are warranted to refine timing and dosing parameters.

The therapeutic mechanisms of platelet-rich plasma (PRP) in the management of thin endometrium are primarily mediated through three distinct yet interrelated pathways:

Enhancement of endometrial cell proliferation and growth: PRP contains a high concentration of growth factors, including platelet-derived growth factor (PDGF), epidermal growth factor (EGF), vascular endothelial growth factor (VEGF), transforming growth factor (TGF), insulin-like growth factor (IGF), and fibroblast growth factor (FGF). These factors act synergistically to activate cell surface signaling pathways such as nuclear factor κB (NF-κB), phosphoinositide 3-kinase (PI3K), platelet-derived growth factor receptor (PDGFR), and protein kinase B (Akt), thereby promoting cellular mitosis. This process facilitates the migration and proliferation of endometrial epithelial cells, endometrial stromal fibroblasts, endometrial mesenchymal stem cells (EnMSCs), and bone marrow-derived mesenchymal stem cells (BMSCs), accelerating the regeneration of the functional endometrial layer. Clinically, this manifests as increased endometrial epithelial cell proliferation, enhanced glandular epithelial density, and improved endometrial growth and differentiation (31–33). Additionally, the fibrinogen in PRP forms a gel upon activation, providing a temporary scaffold for cell proliferation and migration. This gel matrix also protects bioactive factors released from platelets from proteolytic degradation, enabling their sustained release and prolonging their therapeutic effects on target cells (34). Furthermore, PRP significantly upregulates the expression of angiogenesis-related factors such as keratin, homeobox A10 (HOXA10), and VEGF, increasing endometrial glandular density, reducing fibrosis, and promoting collagen synthesis and secretion. These effects collectively enhance tissue repair and improve endometrial receptivity (35, 36).

Promotion of angiogenesis: Thin endometrium is often associated with vascular dysplasia and insufficient blood supply, characterized by elevated uterine artery blood flow impedance, impaired endometrial growth, and reduced VEGF expression (37, 38). The high concentration of growth factors in PRP induces the migration, proliferation, and differentiation of vascular endothelial cells, thereby promoting neovascularization and improving endometrial blood supply. This process optimizes endometrial perfusion and, through the regulation of vascular permeability, creates a favorable microenvironment for successful embryo implantation (39, 40).

Anti-inflammatory effects: Patients with thin endometrium frequently exhibit an inflammatory cascade, particularly involving pro-inflammatory cytokines produced by type 1 helper T cells (Th1), which can compromise endometrial receptivity (41, 42). PRP contains cytokines such as PDGF, FGF, and platelet factor 4 (PF4), which chemoattract macrophages and neutrophils and promote the production of fibrosis-inhibiting matrix metalloproteinases (MMPs), including MMP-3, MMP-7, and MMP-26 (43, 44). PRP also modulates the expression of interleukins (IL)-1α, IL-1β, and IL-10 while suppressing IL-6 production (45). These actions effectively inhibit the release of inflammatory mediators, prevent fibrotic scar formation, and attenuate excessive inflammatory responses in the endometrium, thereby mitigating the progression of endometrial fibrosis.

### Limitations and future perspectives

The current study has several limitations. First, the restricted follow-up period precluded analysis of live birth rates and obstetric outcomes. Second, as a single-center study, our findings require validation through multicenter investigations with larger sample sizes and additional evaluation parameters. Importantly, there remains a lack of consensus regarding optimal PRP preparation protocols, as well as robust evidence supporting the ideal platelet concentration, dosage regimen, and treatment protocol for intrauterine PRP infusion. Therefore, well-designed prospective randomized controlled trials with adequate power are urgently needed to establish standardized PRP preparation and treatment protocols for reproductive applications.

## Conclusion

Compared to a single intrauterine infusion, double intrauterine PRP infusion may enhance the receptivity of a thin endometrium and improve clinical pregnancy outcomes. However, since the study population did not include patients with thin endometrium who also have a history of recurrent implantation failure or recurrent miscarriage, caution is advised when applying these findings to this specific group. Furthermore, these conclusions require validation through larger, randomized, multicenter trials.

## Data Availability

The datasets presented in this study can be found in online repositories. The names of the repository/repositories and accession number(s) can be found below: http://www.medresman.org.cn/login.aspx (ChiCTR2300071473).
